# Histamine binding activity of surface-modified yeast by histamine binding protein (HBP)

**DOI:** 10.1186/s13568-021-01308-3

**Published:** 2021-10-29

**Authors:** Hyeweon Jang, Geun Woo Lee, Yang-Hoon Kim, Jiho Min

**Affiliations:** 1 Graduate School of Semiconductor and Chemical Engineering, Jeonbuk National University, 567 Baekje-daero, Deokjin-gu, 54896 Jeonju-si, Jeollabuk-do Republic of Korea; 2 School of Biological Sciences, Chungbuk National University, Chungdae-Ro, Seowon-Gu, 28644 Cheongju, South Korea

**Keywords:** Histamine binding protein, Recombinant yeast, Histamine, Anti-allergy, Anti-inflammatory

## Abstract

Histamine is an immune mediator that is mainly secreted when an immediate, rapid response is needed in the body, and an excessive secretion of histamine or lack of enzymes that degrade histamine can result in various side effects. Histamine binding protein (HBP) is secreted by a mite species to prevent the host’s histamine-induced immune responses by binding the histamine molecule in the blood. Cloning was performed to express HBP on the yeast surface (MBTL-GWL-1), and immunofluorescence (IF) and western blot was performed to confirm the expression of the recombinant protein. The histamine inhibitory ability of GWL-1 cells was tested according to the cell concentration. The highest inhibitory ability of 1.30 × 10^7^ CFU/ml of GWL-1 cells was of about 60 %. The GWL-1 cell concentration and the degree of histamine inhibition were confirmed to be dose-dependent, and dead cell debris was shown to have a histamine inhibitory effect, although not as much as that of whole cells. Phagocytosis assays were performed to determine whether histamine affected the RAW 264.7 cell’s phagocytosis, and to indirectly confirm the GWL-1 cell’s histamine inhibition. By confirming that, we found that GWL-1 captures histamine. Therefore, it can be expected to become a competitive material in the anti-allergy market.

## Key points

- The new material for histamine inhibitors was made by modifying the yeast surface.
- Confirmation of histamine-binding ability of a new histamine-capturing material.
- Another way to control histamine to relieve excessive inflammation are proposed.

## Introduction

Histamine [2-(4-imidazolyl)-ethylamine] is an immune mediator that is secreted mainly when an immediate, rapid response is needed in the body. When there is an external stimulus, basophils and mast cells secrete histamine, and they start the body’s immune system response. If histamine is normally regulated, it is not a problem, but an excessive secretion of histamine or lack of enzymes that degrade histamine can result in itching and inflammatory reactions (Schneider et al. 2010). In particular, skin diseases that are caused by the action of uncontrolled histamine, such as atopy and psoriasis, are often a problem because this reaction mediates the vasodilatation and skin nerve stimulation that cause itching (Werfel et al. 2016). Therefore, antihistamines are widely used to prevent various allergic reactions by binding to histamine receptors and suppressing the immune response caused by histamine (Dykewicz et al. 1998). However, antihistamines often cause side effects in the central nervous system including drowsiness, fatigue, memory loss, and attention deficit, as well as digestive disorders such as constipation diarrhea, nausea, and vomiting (Hindmarch and Shamsi 1999).

Histamine binding protein (HBP) is secreted by *Rhipicephalus appendiculatus*, a mite species, to prevent the host’s histamine-induced immune response. When the tick bites the host, it injures the wound site and secretes HBP into the host. The HBP entering the host binds to the histamine molecule in the blood and suppresses its activity in the host. As such, HBP inhibits the host’s inflammatory response, helps the ticks adhere to the skin, and aids reproductive success (Paesen et al. 1999).

Phagocytosis is an immune response that takes place inside the body to counter invading substances from the outside (Aderem and Underhill 1999). Phagocytosis is a defense mechanism by cells against foreign substances. Histamine acted as an inhibitor for phagocytosis of RAW 264.7 cells at a high concentration (10^−5^ M) (Azuma et al. 2001; Khan and Rai 2007). Therefore, we have tried to indirectly determine the effect of histamine inhibition through this observation.

This study has developed a histamine binding agent to replace antihistamine, and the histamine binding activity of recombinant HBP on the yeast surface was confirmed in the histamine solution. We need another drug that can only control histamine molecules without reacting with other receptors, like HBP. As such, it can be expected that HBP will become a competitive material in the anti-allergy market.

## Materials and methods

### The recombinant plasmid

The gene sequence of HBP2 (Female-specific Histamine-Binding Protein 2, *Rhipicephalus appendiculatus*, Accesion number U96081) is deposited in the GenBank (NCBI, USA) with accession No. U96081, and it was obtained by requesting gene synthesis from Bioneer (Daejeon, Korea). The gene sequences of Cwp2p (Cell wall protein, the major mannoprotein of the yeast cell wall) is deposited in the GenBank with accession No. NM_001180025, and it was provided by Chungbuk National University (Lee et al. 2017).

The recombinant plasmid was constructed based on the pYES2 vector (Invitrogen) and was synthesized in the order of the Cwp2p signal peptide (60 bp), HA tag (27 bp) and histamine binding protein 2 (513 bp), Gly-Ser linker (30 bp) and Cwp2p gene (219 bp). The signal peptide and the stop codon of the histamine binding protein were excluded from the recombinant plasmid. The synthesized gene from HBP2 was provided in a pBHA vector containing the antibiotic Ampicillin resistance gene. After confirming the concentration of the vector, it was transformed into *Escherichia coli* DH5α to amplify the gene. *E. coli* DH5α cells were provided by the Real Biotech Corporation (Taipei, Taiwan).

### Culture of recombinant *E. coli* and Plasmid isolation

Since the *E. coli* transformed with the synthesized vector contains resistance to ampicillin, it can be selectively cultured using medium containing ampicillin. For amplification of the vector, recombinant *E. coli* was cultured in Luria-Bertani medium (1 % tryptone, 0.5 % yeast extract, 1 % NaCl and sterilization at 121 °C for 15 min) containing 100 µg/ml ampicillin. *E. coli* was incubated overnight at 37 °C at 180 rpm. After culturing, the cells were collected via centrifugation. The plasmids were extracted from the collected cells using Gene all Explin Plasmid SV kit that was provided by GeneAll Biotechnology (Seoul, Korea). The extracted plasmid was identified and was quantified via agarose gel electrophoresis.

### Construction of plasmid DNA pYES2::CSP::HBP2::Cwp2p

The gene was cut with restriction enzymes and was ligated to insert DNA and vector DNA using the T4 ligase. The restriction enzymes and T4 ligase were provided by Enzynomics (Daejeon, Korea). The completed plasmid was precipitated via ethanol precipitation and was transformed into *E. coli* for amplification. The completed recombinant *E. coli* was incubated overnight at 180 rpm at 37 °C, and the plasmid was extracted.

The completed plasmid was subjected to a sequencing analysis using two sequencing primers of HBP2 seq F-552 (5’-TGA GTA CGC TGT AGG TAG GG) and HBP2 seq R-658 (5’-ATG GCG GCA GCG GAT TCA GA-3 ‘). Sequence analyses were performed via Cosmo Genetech (Daejeon, Korea).

### Transformation in yeast *S. cerevisiae*

The completed plasmid was subjected to yeast transformation using the lithium acetate method (Schiestl and Gietz 1989), and the *S. cerevisiae* 2805 strain (ATCC 208,280) was used for yeast transformation. *S. cerevisiae* strains were grown in Yeast Extract-Peptone-Dextrose (YPD) medium (1 % yeast extract, 2 % peptone and 2 % D-glucose) until they had an optical density (OD) 600 of 0.9–1.0, and 1.5 ml of the strains were collected via centrifugation. Next, 1 ml of 0.2 M lithium acetate was added and washed. Then, the cells were collected via centrifugation again. 20 µL of 1 M lituuim acetate, 10 µL of plasmid and 30 µL of 70 % polyethylene glycol were added to the collected cells, followed by incubation for 1 h at 30 °C and 180 rpm. Next, distilled water (DW) 140 µL was added and 100 µL cells were spread on synthetic dextrose (SD) solid medium (0.67 % yeast nitrogen base, 0.5 % casamino acid, 2 % D-glucose, and 1.5 % agar) to select transformed yeast.

The recombinant yeast in which HBP was expressed on the yeast surface was named GWL-1 cells (Cwp2p signal peptide :: HA tag :: HBP :: Gly-Ser linker :: Cwp2p :: pYES2). In addition, as a control, GWL-0 cells (Cwp2p signal peptide :: HA tag :: Gly-Ser linker :: Cwp2p :: pYES2) and Mock with only the pYES2 vector were transformed in the yeast.

### Recombinant yeast culture and induction

Mock and recombinant strains were each grown on SD minimal medium (0.67 % yeast nitrogen base, 0.5 % casamino acid and 2 % D-glucose) until OD600 0.7 at 30 °C and 180 rpm. The grown cells were centrifuged at 3000 rpm for 5 min, and the supernatant was then removed. To induce the GAL promoter of pYES2 vector, the collected cells were suspended in synthetic galactose (SG) medium (0.67 % yeast nitrogen base, 0.5 % casamino acid and 2 % D-galactose) and were cultured until OD600 0.78 at 30 °C for 20 h at 180 rpm.

### Histamine reduction ability test (LC–MS)

The cells were grown in SD medium to OD600 0.7 for the histamine inhibitory ability of the recombinant yeast. The cells were collected by centrifugation, and the medium was replaced by SG medium, followed by induction for 20 h. The induced cells were cultured until OD600 0.78. The amount for 10 ml of induced cells was 1.30 × 10^7^ CFU. Also, the cells were homogenized by a sonicator (SONICS & MATERIALS INC. 53 CHURCH HISLL RD. NEWTOWN, CT. U.S.A) and were tested to see if they could affect histamine even without living whole cells. Whole cells and cell debris were prepared by centrifugation and washing with DW. The whole cells and the cell debris were reacted with 1 ml of histamine solutions of various concentrations followed by a slow rotation for 2 h. After the rotation, whole cells and cell debris were centrifuged, and the supernatant was filtrated to separate cells from the unreacted histamine solution. The histamine solutions for which the reaction was complete were quantitated via LC–MS analyses (Center for University-Wide Research Facilities, Jeonbuk National University, Korea).

### Western blot

The cell debris was prepared by homogenizing the induced cells, and the protein lysate was prepared using NP-40 buffer (150 mM NaOH, 1.0 % NP-40, 50 mM tris (pH 8.0)) containing protease and phosphatase. Their protein concentrations were quantitated using Quick Start Bradford 1X Dye reagent (Bio-rad). The samples were run on a 15 % SDS-PAGE, transferred to nitrocellulose membranes, and were then blocked with 3 % bovine serum albumin in T-TBS (20 mM Tris, 150 mM NaCl, 0.1 % Tween 20 detergent). The membranes were incubated overnight at 4 ℃ with Anti-HA tag (abcam, 1:500 dilution). The blots were washed and incubated for 1 h at room temperature with the Gt anti-Ms IgG (H+L) secondary antibodies (Invitrogen, 1:5000 dilution). Immunoreactive protein bands were visualized with ECL Prime Western Blotting Detection Reagents (Amersham) and light emission detected by exposing the membrane to X-ray film (AGFA).

### Immunofluorescence

The induced cells that were adjusted to OD600 0.5 were collected in 10 ml samples. The collected cells were washed 2 times using 1X Phosphate-buffered saline (PBS, NaCl 137 mM, KCl 2.7 mM, Na_2_HPO_4_ 10 mM, KH_2_PO_4_ 1.8 mM) containing protease, blocked 1 h with 3 % bovine serum albumin in 1X PBS, incubated overnight at 4 ℃ with Anti-HA tag (abcam, 1:500 dilution), washed 5 times using 3 % BSA in 1X PBS containing protease, incubated for 1 h at room temperature with m-IgG BP-FITC (Santa Cruz Biotechnology, 1:200 dilution) and washed using 1X PBS containing protease. The immunized cells were observed using an Axioscope (Carl Zeiss).

### Animal cell culture

The murine macrophage RAW 264.7 cells were purchased from the Korean cell line bank (KCLB) and were cultured in Dulbecco’s Modified Eagle’s Medium (DMEM, GIBCO), supplemented with 10 % Fetal Bovine Serum (GIBCO), 100 U/mL penicillin, and 100 µg/mL streptomycin in a humidified 5 % CO_2_ atmosphere at 37 °C.

### MTT assay

The cytotoxicity of the histamine to RAW 264.7 cells was confirmed by an MTT assay. The cells adjusted to 1 × 10^5^/ml were cultured for 24 h and starved for 12 h. The histamine concentration in normal human’s whole blood is 2.5–6.5 × 10^−2^ ppm (Rirodan et al. 1998). Therefore, the histamine concentration used in this experiment was set to 10^−2^ – 10 ppm. The histamine solution was treated based on 1X PBS for 2 h at 37 °C. MTT stock (50 mg/ml Thiazolyl Blue Tetrazolium Bromide) was diluted by free-serum DMEM and treated to RAW 264.7 cells for 2 h. Dimethyl sulfoxide (DMSO) was treated and the absorbance was read at 540nm by a spectrophotometer. Then, the cell viability was calculated.

### Phagocytosis assay

Phagocytosis assays were performed by a product manual provided by CELL BIOLABS. First, various concentrations of histamine solution were used that reacted and did not react with the induced GWL-1 cells. RAW 264.7 cells were adjusted to 5 × 10^5^/ml, cultured for 24 h, and starved for 12 h. The histamine solution was prepared at 0.1, 1, 10 and 100 ppm based on 1X PBS. Then, the histamine solutions were reacted with GWL-1 cells 1.30 × 10^7^ CFU/ml by a rotator for 2 h and centrifuged, and then the supernatant was filtrated. RAW 264.7 cells were preincubated with a histamine solution that was diluted 1/10 with final concentrations of 0.01, 0.1, 1 and 10 ppm for 2 h. Zymosan suspension provided by the phagocytosis assay kit was used as the phagocytosis stimulant. To measure the degree of phagocytosis, the absorbance was detected at 405 nm by a spectrophotometer, and the phagocytosis activity was calculated.

### Data analysis

Each data point was obtained from three independent samples analyzed simultaneously to conduct error analysis. The averages are reported with the standard deviations and correlations for several experimental conditions. The data were analyzed using SigmaPlot (Systat Software, Inc., USA). A p-value < 0.05 was considered significant.

## Results

### Construction of the recombinant yeast

Female-specific histamine-binding protein 2 derived from *Rhipicephalus appendiculatus* was expressed on the yeast surface. Cwp2 yeast membrane receptors and signal peptides were used to express HBP on the yeast surface. Cloning was performed by inserting the HBP protein between the Cwp2 membrane protein and the signal peptide. Gly-Ser linker was added to prevent the protein from being modified and HA tag was added to check the protein expression. We refer to these cells as MBTL-GWL-1. To compare the effect on the recombinant cells, GWL-0 that excluded only the HBP gene from GWL-1’s vector and a Mock with only the pYES2 vector were used as controls.

### Confirmation of HBP expression

#### Immunofluorescence

**Fig. 1:**
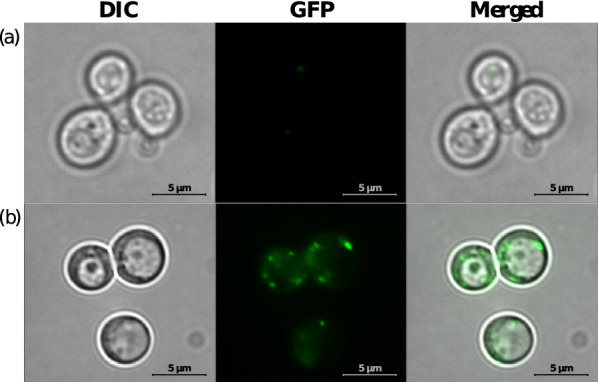
Immunofluorescence with Mock vector. **a** Mock vector. **b** GWL-1 cells. To confirm the expression of recombinant protein, we performed Immunofluorescence. A fluorescent secondary antibody was attached to the recombinant protein of the induced cells. As a control, Mock was used. While the control did not show fluorescence, fluorescence was successfully expressed in GWL-1 cells

A fluorescent secondary antibody was attached to the recombinant protein of the induced cells. Mock was used as control. As shown in the Fig. 1, control did not show fluorescence. However, the GWL-1 cells showed green fluorescence, and the expression of the recombinant protein was successfully confirmed in the GWL-1 cells.

#### Western blot

**Fig. 2:**
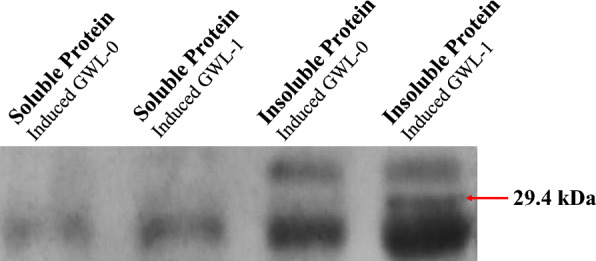
Western blot was performed to confirm expression of the recombinant protein. Cells were sonicated and separated into soluble and insoluble proteins. Since HBP was expressed on the cell surface, it is an insoluble protein, and the protein size of recombinant protein is 29.4 kDa. As a control, GWL-0 cells were used expressing a yeast surface protein (Cwp2p) without the HBP gene. As shown in the Fig. 4, a band of 29.4 kDa is seen in the insoluble protein of the GWL-1 strain

The induced cells were sonicated and separated into soluble and insoluble proteins. HBP was expressed as a surface protein, and the expressed protein is an insoluble protein with a protein size of about 29.4 kDa. As a control, GWL-0 cells were used. The GWL-0 strain expressed a yeast surface protein (Cwp2p) without the HBP gene. As shown in the Fig. 2, a band of 29.4 kDa is seen in the insoluble protein of the GWL-1 strain.

### Histamine reduction ability test (LC–MS)

#### Cell concentration

**Fig. 3:**
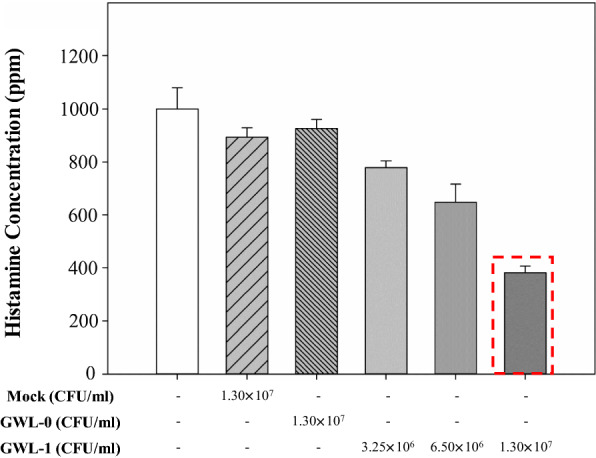
Histamine inhibitory ability according to the cell concentration. GWL-1 cells were tested by the cell concentration. Mock and GWL-0 cells were used as controls. The histamine inhibitory ability of GWL-1 was better than that of controls, and 1.30 × 10^7^ CFU/ml of GWL-1 showed the highest inhibitory ability compared to other cell concentrations. The GWL-1 cell concentration and the degree of histamine inhibition were confirmed to be dose-dependent

The induced cells were tested by the cell concentration, and Mock vector, MBTL-GWL-0 strain were used as controls. As shown in Fig. 3, the histamine inhibition of GWL-1 was better than that of the controls, and GWL-1 cells at 1.30 × 10^7^ CFU/ml showed the highest inhibitory ability compared to other cell concentrations. The GWL-1 cell concentration and the degree of histamine inhibition were confirmed to be dose-dependent.

#### Sonicated cell

**Fig. 4:**
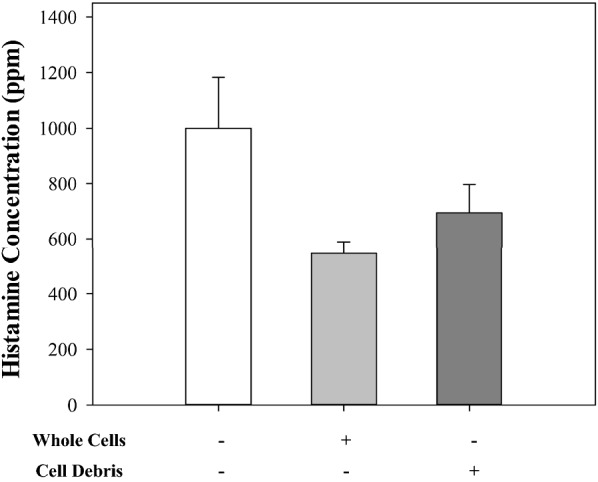
Histamine inhibitory ability in cell debris. The cells were sonicated and reacted with histamine to confirm their inhibitory ability. The result confirmed that the cell debris of GWL-1 also showed a histamine inhibition as much as for the whole cells

Homogenized cells were tested via LC/MS with live GWL-1 cells. As confirmed in Fig. 4, the cell debris of GWL-1 also showed histamine inhibition as much as the whole cells.

### Phagocytosis assay

**Fig. 5:**
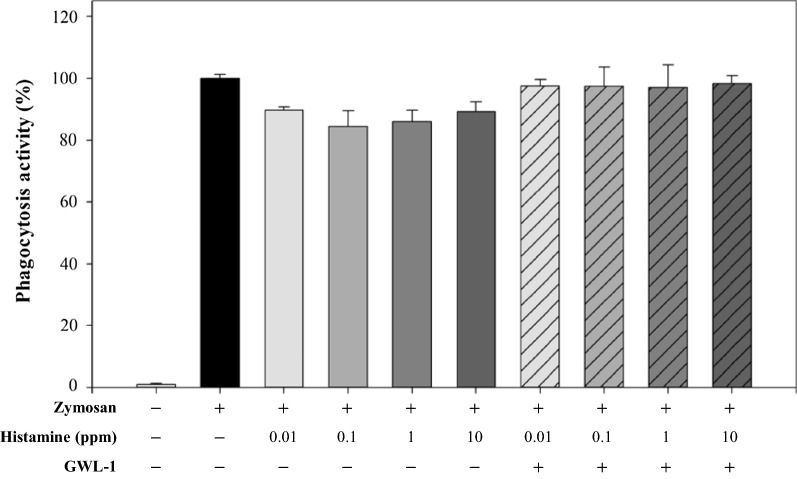
Phagocytosis activity. Phagocytosis assays were performed to determine whether histamine affected RAW 264.7 cell’s phagocytosis, and to confirm the GWL-1 cell’s histamine inhibitory ability indirectly. Histamine inhibited the phagocytosis of RAW cells, and histamine reacting with GWL-1 did not inhibit phagocytosis. As a result of confirming this, GWL-1 was found to capture histamine

Before the phagocytosis assay, MTT assays of RAW 264.7 cells for histamine were performed. The results indicated no cytotoxicity at the given histamine concentration (10^−2^ – 10 ppm) in the analysis (Data not shown). The phagocytosis assay was used to determine whether histamine affected RAW 264.7 cell phagocytosis, and it was used to confirm the histamine inhibition of the GWL-1 cells. Histamine acted as an inhibitor for the RAW 264.7 cell’s phagocytosis at a high concentration (10^−5^ M) (Azuma et al. 2001; Khan and Rai 2007). The histamine solution was prepared without reaction with GWL-1 cells and was prepared by a reaction with GWL-1 cells. The prepared histamine solutions were treated with RAW 264.7 cells. As shown Fig. 5, phagocytosis of the RAW 264.7 cells treated with unreacted histamine solution was meaningfully reduced. In addition, the phagocytosis of RAW 264.7 cells treated with the histamine solution reacted with GWL-1 cells recovered compared to that of the histamine solution not treated GWL-1. This confirmed that GWL-1 can capture histamine molecules.

## Discussion

Antihistamines are usually prescribed for allergy symptoms (Dykewicz et al. 1998), and other anti-allergy substances are actively being studied. Despite the side effects of the antihistamines (Schinella et al. 2002; Makchuchit et al. 2017; Park et al. 2017), there are no alternatives to antihistamine yet. Therefore, we need to develop other anti-allergy substance from biomolecules. Yeast is a familiar microbe that is also uses in food (Halász and Lásztity 2017). It has the advantage of being able to be mass-produced because its incubation conditions are not difficult (Brochado et al. 2010). In addition, HBP is a substance secreted by mites to hosts, and it is known to regulate histamine in the host’s body (Paesen et al. 1999).

HBP can be mass-produced by cloning the yeast, and it can be considered for use as the cell itself through display on the yeast surface. The expressed protein was confirmed via Western blot (WB) and Immunofluorescence (IF). This confirmed the use of the proteins expressed on the yeast surface. It confirmed the ability to capture the histamine molecule itself on the cells, and the use of the protein on the whole cells could be considered. It is believed that this has a positive effect since no other process step is required. However, since genetically engineered organisms can be subjected to rejection, we investigated their ability to reduce histamine even in cell debris. It is as effective as whole cells in cell debris, so it seems that it can be used for various purposes.

Phagocytosis is an immune response that takes place inside the body to counter invading substances from the outside. Histamine acted as an inhibitor of RAW 264.7 cell phagocytosis at high concentrations (10^−5^ M) (Azuma et al. 2001; Khan and Rai 2007). Therefore, we indirectly confirmed the effect of the inhibition of histamine by GWL-1 using the results of the phagocytosis assay. The results indicated a meaningful reduction in the phagocytosis of RAW 264.7 cells treated with unreacted histamine solution. The phagocytosis of RAW 264.7 cells treated with the histamine solution reacted with GWL-1 cells recovered compared to that of the histamine solution not treated with GWL-1. Through phagocytosis assay, it was confirmed that GWL-1 cells could effectively inhibit histamine. The purpose of this experiment is to develop a histamine inhibitor to counter the histamine molecules, which is meaningful in that it is approached in a different way from that of existing materials. The current histamine inhibitor in use, antihistamines, inhibit the behavior of histamines by binding to histamine receptors, and antihistamines bind not only to histamine receptors but also to other receptors, so they have various side effects (Hindmarch and Shamsi 1999). However, the subject of this study inhibits the behavior of histamine by directly capturing histamine molecules, and the substances in this study do not bind to other receptors, so they can be expected to have positive effects.

This study can be helpful toward improving consumer awareness in that it is a yeast-based material that is also used as an ingredient in food (Halász and Lásztity 2017). In addition, it can be used as the cell itself expressing HBP on its surface, so a separate process is not required before use. The fact that it is a recombinant strain can lead to negative recognition, so the inhibition of the histamine in cell debris was also confirmed. This study confirmed that histamine inhibitors capture histamine molecules, so they can be used by considering countermeasures against various side effects for antihistamine in the future.

However, this study had not yet been directly processed in vitro, and a viability test for this material must be performed. Also, since in vivo experiments were not performed, the safety of the material in this study should also be confirmed.

Nevertheless, in the study, the material has many advantages in that it can replace the side effects of the current histamine inhibitors. Therefore, if it is developed through follow-up research, it will be implemented to great effect as a histamine inhibitor.

## Data Availability

The gene sequences used for yeast surface expression of HBP and Cwp2p can be searched in NCBI GenBank database with the accession number U96081 and NM_001180025. The strain of *S. cerevisiae* 2805 used for transformation and protein expression is ATCC 208,280.

## Abbreviations

HBP: Histamine binding protein
YPD: Yeast extract-Peptone-Dextrose
OD: Optical density
DW: Distilled water
SD: Synthetic dextrose
SG: Synthetic galactose
CFU: Colony forming unit
LC-MS: Liquid chromatography-mass spectrometry
NP-40: Nonidet P-40
SDS-PAGE: Sodium dodecyl-sulfate polyacrylamide gel electrophoresis
TBS: Tris-buffered saline
PBS: Phosphate-buffered saline
DMEM: Dulbecco’s Modified Eagle’s Medium
MTT: 3-(4,5-Dimethylthiazol-2-yl)-2,5-diphenyltetrazolium bromide
